# Lowering the osteotomized level of fibular osteotomy reduces neuromuscular complications while maintaining clinical efficacy in treating medial compartment knee osteoarthritis: a retrospective comparative cohort study

**DOI:** 10.1186/s40001-025-02919-3

**Published:** 2025-07-24

**Authors:** Ting-Yu Chang, Chih-Wei Chang, Yen-Nien Chen, Chyun-Yu Yang, Jou-Hua Wang

**Affiliations:** 1 Department of Orthopedics, Taipei Veterans General Hospital, Taipei, Taiwan; 2 Department of Orthopedics, College of Medicine, National Cheng Kung University, No.138, Sheng Li Road, Tainan, 704 Taiwan; 3 Department of Orthopedics, National Cheng Kung University Hospital, College of Medicine, National Cheng Kung University, Tainan, Taiwan; 4 Department of Orthopedic Surgery, Kuo General Hospital, Tainan, Taiwan; 5 Department of Physical Therapy, Asia University, Taichung, Taiwan

**Keywords:** Partial fibular osteotomy, Knee osteoarthritis, Peroneal neuropathy, Osteotomized level, Complication

## Abstract

**Background:**

Partial fibular osteotomy (PFO) offers a minimally invasive strategy for treating medial compartment knee osteoarthritis (MKOA), yet its proximity to the peroneal nerve raises concern for postoperative neuropathy. This study investigated whether lowering the osteotomy site from the upper third to the mid-third of the fibula reduces nerve complications without compromising clinical efficacy.

**Methods:**

We retrospectively reviewed 77 consecutive patients who underwent unilateral PFO from March to December 2018. After excluding 20 patients due to prior limb surgeries, or neurological comorbidities, inadequate follow-up or incomplete records, 57 patients (61 knees) with Kellgren–Lawrence grade II–III MKOA were included. Patients were allocated chronologically, with the surgical protocol modified in August 2018 after observing high neuropathy rates in the initial cohort. The osteotomy level was adjusted from 6–10 cm (upper group) to 12–15 cm (lower group) below the fibular head following early observed neuropathies. Outcomes included Oxford Knee Score (OKS), Visual Analog Scale (VAS) for pain, femorotibial angle (FTA), medial joint space ratio (MJSR), and peroneal neuropathy incidence. Radiographs were taken at baseline and 6-month follow-up. Between-group comparisons used independent *t* tests and Mann–Whitney *U* tests for continuous variables, and chi-square tests for categorical outcomes. Although a priori power analysis was performed—a limitation of this retrospective design—post hoc calculations confirmed adequate effect size detection. Mean follow-up was 13.4 months (range 12–15).

**Results:**

Demographics were similar between groups (e.g., mean age 64.0 vs. 62.6 years, *p* = 0.67). OKS improved from 23.2 ± 5.2 to 37.7 ± 3.3 in the upper group and from 27.0 ± 8.9 to 38.1 ± 6.0 in the lower group. Over 85% of patients achieved a ≥ five-point OKS improvement, a threshold representing a clinically meaningful difference. VAS scores decreased to 1.8 ± 0.8 and 1.9 ± 0.8, respectively (*p* < 0.001) Peroneal neuropathy occurred in 37.9% (toe weakness) and 31.0% (numbness) of upper-group knees, with no deficits observed in the lower group (*p* < 0.05). Radiographic alignment and joint space changes were minimal across both cohorts.

**Conclusions:**

Lowering the osteotomy to the mid-third of the fibula significantly reduced peroneal nerve injury while preserving functional improvement. These findings support mid-level PFO as a safer, technically accessible modification for MKOA treatment.

## Introduction

Knee osteoarthritis (OA), particularly in the medial compartment, is a major source of pain and disability in aging populations. While high tibial osteotomy (HTO) and total knee arthroplasty (TKA) remain gold-standard interventions, not all patients are ideal candidates for these procedures due to cost, invasiveness, or comorbidity profiles [1, 2].

Partial fibular osteotomy (PFO), originally described as an adjunct to HTO to facilitate tibial realignment [3], has more recently gained traction as a standalone procedure for medial knee osteoarthritis (MKOA). It is typically indicated for patients with medial compartment OA, mild-to-moderate varus deformity, and an intact lateral compartment—particularly those who are ineligible for or reluctant to undergo more invasive procedures, such as HTO or total knee arthroplasty (TKA). In this context, PFO has emerged as a minimally invasive, cost-effective alternative, especially in East and Southeast Asia [4–7]. However, its adoption remains limited in Western countries, in part due to concerns about its mechanism of action and complication profile.

The procedure is thought to improve medial compartment unloading through several proposed mechanisms: lateral column shortening, valgus realignment, release of tibiofibular arch tension (the “arch-string theory”), and dynamic redistribution of load during gait [8–14]. Despite favorable clinical outcomes, the dominant biomechanical mechanism remains a subject of debate. For instance, finite element analysis (FEA) by Kang et al. demonstrated that fibular segment removal at various levels consistently reduces medial compartment stress without producing significant coronal alignment correction [8]. This may explain why some studies report radiographic changes postoperatively, while others—including ours—do not.

Traditionally, PFO is performed 6–10 cm below the fibular head [4, 5]. However, this region coincides with the superficial course of the common peroneal nerve, which typically wraps around the fibular neck 2–4 cm below the fibular head before bifurcating into its superficial and deep branches. This anatomical proximity increases the risk of iatrogenic nerve injury. Published complication rates for peroneal neuropathy following PFO range from 0% to 11.9% [4–6, 12, 15–17], with symptoms, including toe drop, foot numbness, and sensory disturbances. To mitigate this risk, our institution revised the surgical protocol in mid-2018, lowering the osteotomy site to the mid-third of the fibula (12–15 cm distal to the fibular head), aligning with anatomical studies that identify this region as a safer surgical corridor [18–20]. This technical modification was driven not by a desire for greater minimal invasiveness, but by a patient safety imperative: to reduce neurological complications without compromising treatment efficacy. Yet, few clinical studies have directly compared outcomes based on osteotomy level, leaving a critical gap in the literature regarding whether osteotomy height impacts both complication rates and therapeutic benefit.

This study aimed to address two primary questions: (1) does lowering the fibular osteotomy level reduce the incidence of peroneal neuropathy? and (2) Does this modification preserve clinical outcomes—including pain relief (Visual Analog Scale), functional improvement (Oxford Knee Score), and radiographic stability (femorotibial angle and medial joint space ratio)—relative to conventional PFO? By integrating anatomical rationale, clinical outcomes, and procedural safety, we aim to clarify whether this simple technical adjustment can enhance the safety and consistency of PFO in treating MKOA.

## Materials and methods

### Study design

This was a retrospective observational cohort study designed to evaluate the clinical and radiographic outcomes following partial fibular osteotomy (PFO) in patients with medial compartment knee osteoarthritis (MKOA). The study protocol was approved prospectively by the institutional review board prior to data analysis. (IRB No. B-ER-107-332). The requirement for separate consent for this retrospective analysis was waived due to anonymized data handling. All patients provided written informed consent for surgery.

Electronic medical records and radiographs were reviewed for all consecutive adult patients who underwent PFO between March and December 2018 at our institution*.* Initially, osteotomy was performed 6–10 cm below the fibular head based on published guidelines [4, 5]. However, after increased reports of postoperative neuromuscular symptoms during the first 5 months, the protocol was modified in August 2018 to perform the osteotomy 12–15 cm below the fibular head, consistent with anatomical safe zone recommendations [3, 18–20]. Patients were stratified into two cohorts accordingly: the upper PFO group (6–10 cm) and the lower PFO group (12–15 cm). Although this was an exploratory study, the incidence of peroneal neuropathy was retrospectively designated as the primary outcome due to its direct relevance to the surgical modification.

Post hoc power analysis using *G* Power (v3.1) indicated that with 61 knees, the study had > 80% power to detect a medium effect size (Cohen’s *d* = 0.65) for between-group differences in complication rates at *α* = 0.05.

### Patient selection

#### Inclusion criteria:

- Age ≥ 18 years
- Symptomatic medial compartment knee pain
- Radiographic evidence of MKOA (Kellgren–Lawrence grade II or III) on weight-bearing anteroposterior radiographs [21] without significant lateral or patellofemoral involvement, as assessed by joint space narrowing, osteophyte formation, or subchondral sclerosis on AP, lateral, and skyline views
- Ineligibility or unwillingness to undergo HTO or TKA due to concerns about bone resection, implants placement, prolonged recovery, poor bone quality, or anxiety about revision surgery

#### Exclusion criteria:

- Age ≥ 80 years
- Any lifetime trauma or surgery involving the ipsilateral limb
- Neurological deficits on the affected side secondary to spinal radiculopathy, peripheral neuropathy, or prior cerebrovascular accident
- Inadequate follow-up duration or missing outcome data

**Fig. 1:**
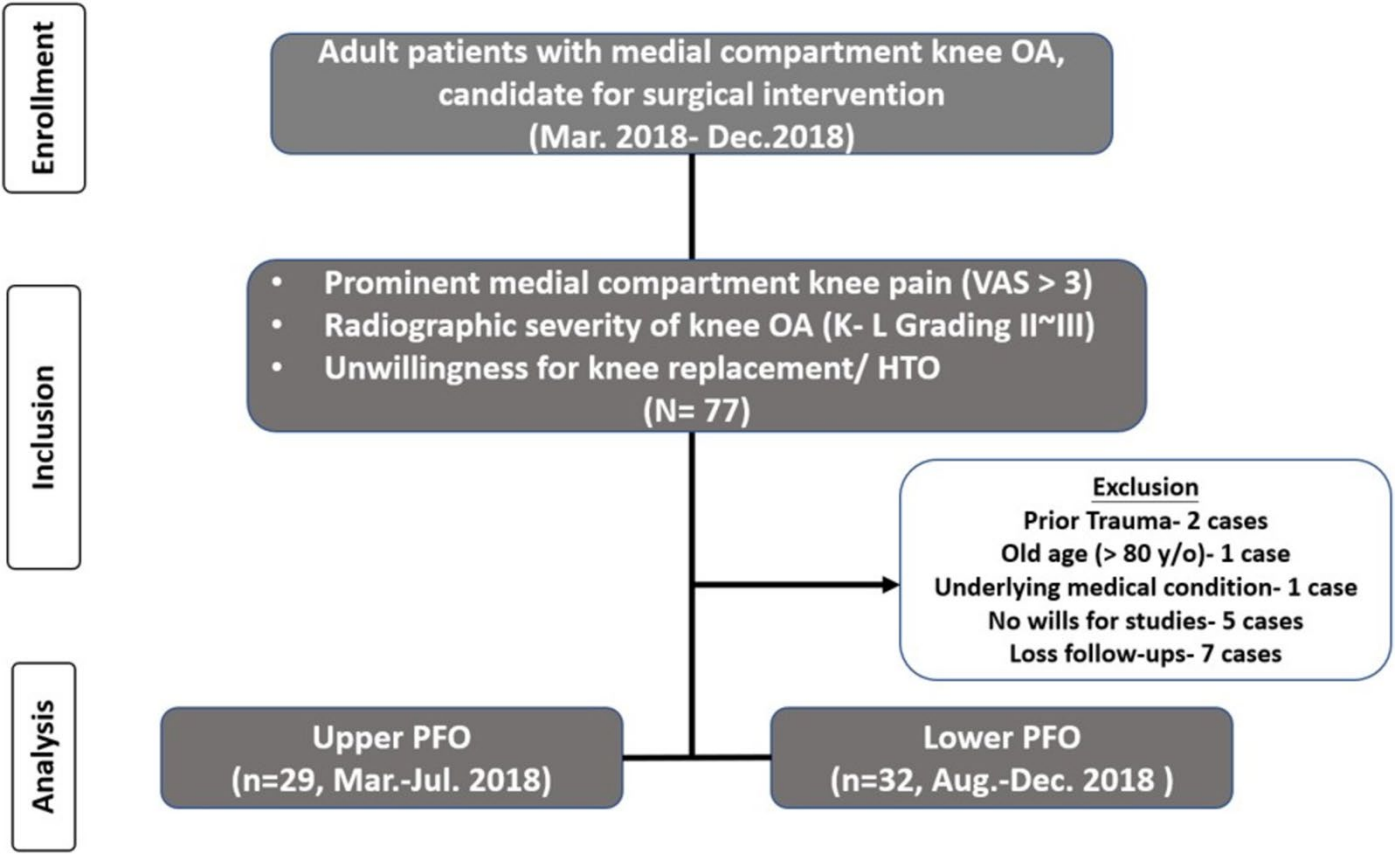
Flowchart of patient enrollment. *VAS* visual analog scale, *K–L* Kellgren–Lawrence, *HTO* high tibial osteotomy, *PFO* partial fibular osteotomy

A visual flowchart of patient enrollment and follow-up compliance is shown in Fig. 1.

### Surgical technique

All procedures were performed by a senior orthopedic surgeon with over 20-year experience, specializing in knee surgeries. Anesthesia (spinal or general) was selected per patient needs, and 1 g IV cefazolin was given preoperatively. Patients were positioned in the lateral decubitus position with the operative knee slightly flexed. Under tourniquet control, a longitudinal posterolateral incision (6–8 cm) was made over the fibula. Blunt and careful dissection was performed between the peroneus longus and soleus to expose the fibular shaft. A 1.5–2 cm fibular segment was resected using an oscillating saw under saline irrigation. Bone wax was applied to osteotomy surfaces to manage bleeding, and wounds were irrigated and closed in layers without drainage. A compressive dressing was applied immediately postoperatively. Group classification was based solely on the osteotomy level, which corresponded to the date of surgery: 6–10 cm from the fibular head prior to August 1, 2018, and 12–15 cm thereafter.

**Fig. 2:**
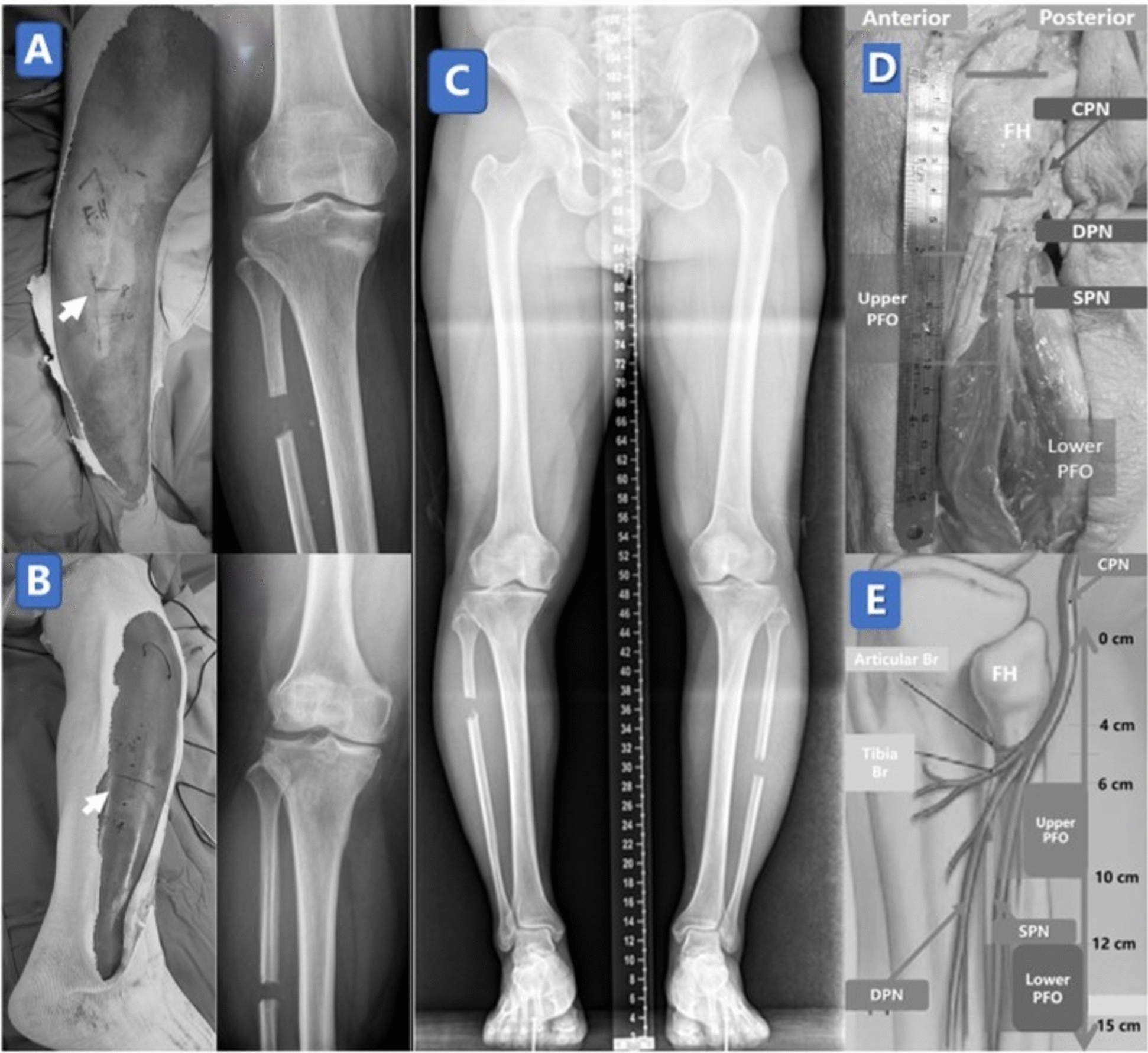
PFO at different osteotomy level and relations to surrounding nerves. **A**, **B** Intraoperative views of upper-level (**A**) and lower-level (**B**) partial fibular osteotomy (PFO), performed 6–10 cm and 12–15 cm distal to the fibular head, respectively. **C** Standing radiograph of a 63-year-old man who underwent staged bilateral PFOs at different levels, showing comparable postoperative alignment and outcomes. **D** Cadaveric dissection highlighting the fibular head (FH), common peroneal nerve (CPN), superficial peroneal nerve (SPN), and deep peroneal nerve (DPN) in relation to osteotomy levels. **E** Schematic depicting upper- and lower-level PFO relative to peroneal nerve branching zones, illustrating the increased neurological safety of the mid-diaphyseal approach

Figure 2 illustrates the imaging differences between upper and lower osteotomy heights and highlights the anatomical relationship between the peroneal nerve and each osteotomy site.

### Postoperative care

Patients began weight-bearing as tolerated on postoperative day 1 with assistive support. Rehabilitation included ankle pumps and quadriceps sets on day 0, followed by progressive weight-bearing, range-of-motion exercises, and stair training under physiotherapist supervision. Most patients achieved independent ambulation by days 1–2. Pain was managed with scheduled NSAIDs and acetaminophen, with tramadol or morphine as rescue anesthesia. Thromboprophylaxis included ankle pumping, early mobilization and mechanical compression, while pharmacologic agents were not routinely used. Discharge criteria included independent ambulation, tolerable pain control with oral medications, and absence of wound complications. Regular follow-ups were scheduled at 2, 6, and 12 weeks, and 6 months postoperatively.

Following surgery, neurosensory complaints—such as numbness, tingling, or muscle weakness—were documented, managed conservatively with pain control, physical therapy, neurologic consultation, and temporary ankle–foot orthosis (AFO) use when appropriate, and prompted targeted clinical evaluations during routine follow-ups.

### Outcome assessments

This study employed a multidimensional outcome assessment strategy to explore clinical efficacy and safety across several domains. Clinical outcomes were assessed at 3 months to capture early functional recovery, while radiographic changes were evaluated at 6 months to allow sufficient time for structural adaptation.

### Clinical outcomes

**Fig. 3:**
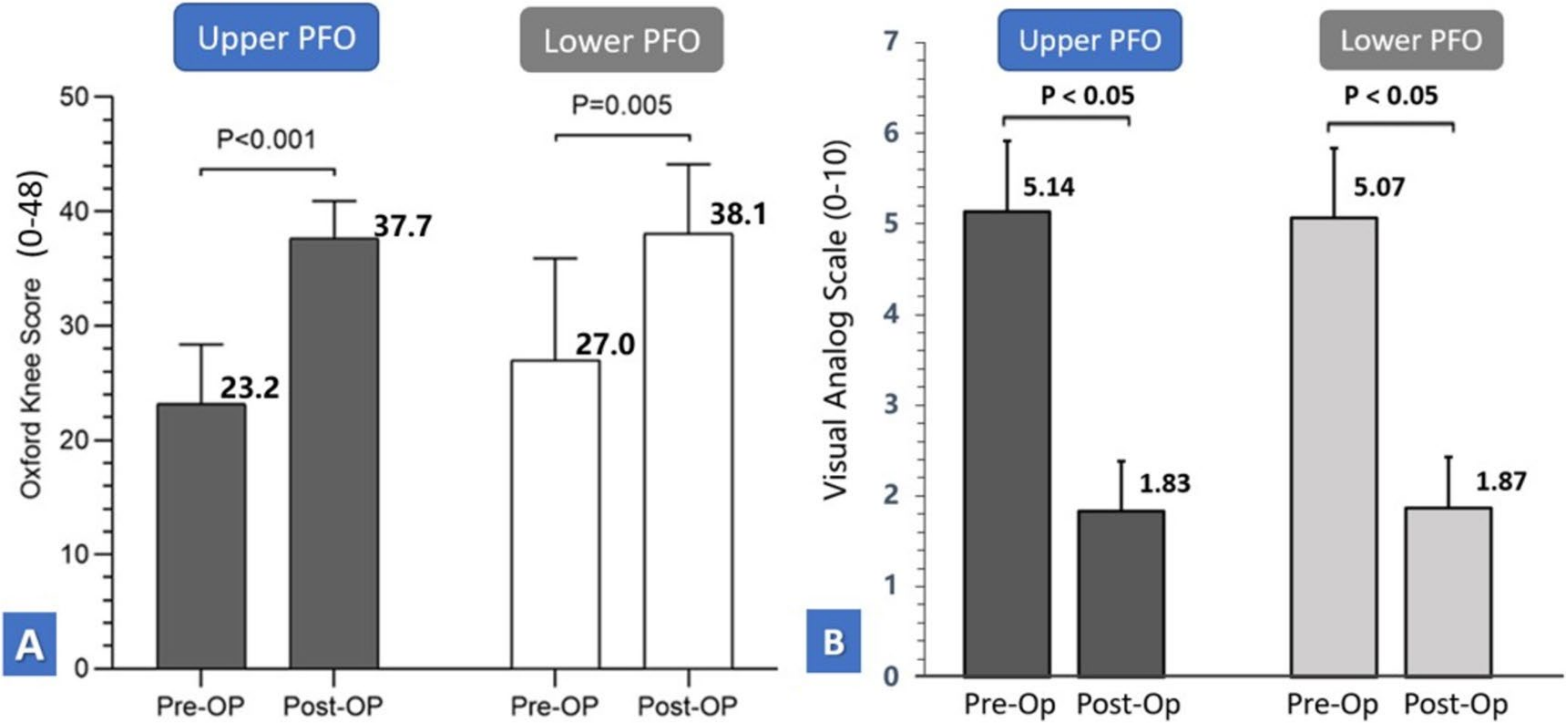
Comparison of pre- and postoperative clinical outcomes following PFO. **A** Oxford Knee Score (OKS); **B** Visual Analog Scale (VAS) for pain. Both upper-level (dark gray) and lower-level (light gray) PFO groups demonstrated significant postoperative improvements in function and pain (*p* < 0.05 vs. baseline). No statistically significant differences were observed between groups postoperatively. Error bars represent standard deviations; bars labeled with * indicate within-group significance (*p* < 0.05)

Functional improvement and pain relief were evaluated using the Oxford Knee Score (OKS) and the Visual Analog Scale (VAS), measured preoperatively and at 3 months postoperatively. Clinical outcome assessors were blinded to group allocation to minimize bias. As no procedure-specific minimal clinically important difference (MCID) has been established for PFO, a threshold of > 5 points in OKS was adopted to represent clinically meaningful improvement, based on MCID estimates from recent studies involving knee osteoarthritis-related surgeries [22, 23] (Fig. 3)

### Neurological and procedural safety

Postoperative neuromuscular safety was assessed at 1, 3, and 6 months by a blinded clinician. Neurologic evaluations included standardized manual muscle testing (MMT) of ankle dorsiflexion, hallux extension, and foot eversion. Strength was graded on a 0–5 scale. Sensory disturbances such as numbness, tingling over the dorsum of the foot or anterolateral shin were mapped using light touch and pinprick testing. Persistent deficits at 6 months without improvement were followed with Electromyography (EMG).

Non-neurologic complications were defined as wound infection, hematoma, dehiscence, or thromboembolic events occurring within 30 days postoperatively.

### Radiographic outcomes

Standardized weight-bearing radiographs were taken in standing full-extension position and slight knee flexion 30 degrees for anteroposterior (AP) and lateral projection, respectively, by trained technicians. Patients stood with knees fully extended and feet shoulder-width apart for AP views, with the X-ray beam centered at the joint line and a standardized source-to-image distance of 100 cm.

**Fig. 4:**
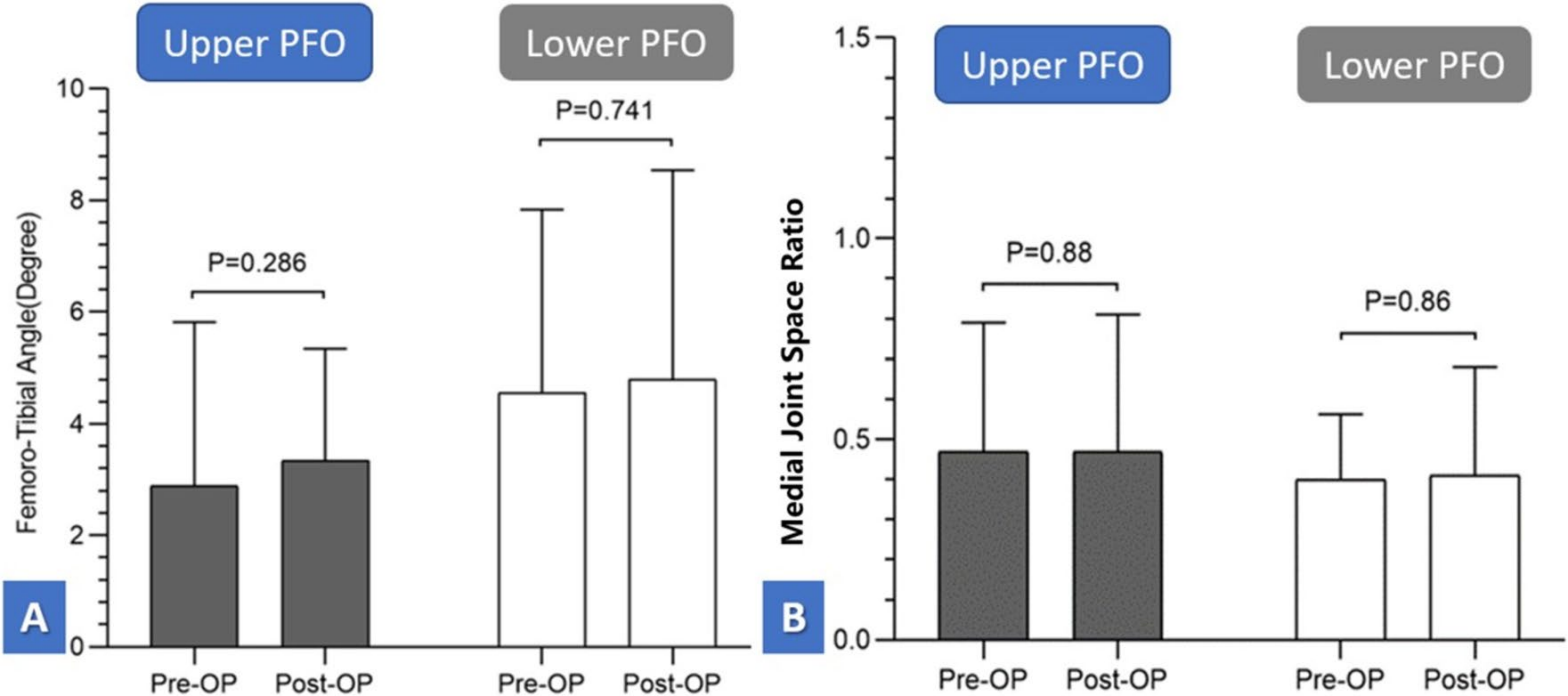
Radiographic outcomes before and after PFO at different levels. **A** Femorotibial angle (FTA); **B** Medial joint space ratio (MJSR). Both parameters were measured in the coronal plane on standardized standing anteroposterior radiographs. FTA was defined as the angle between the anatomical axes of the femur and tibia; values below 5° were considered indicative of residual varus alignment. MJSR was calculated as the vertical height of the medial tibiofemoral compartment divided by the total tibial plateau width. Both upper-level and lower-level PFO groups exhibited minimal changes postoperatively. Slight valgus shifts in FTA and modest variations in MJSR were observed, but none reached statistical significance (*p* > 0.05 in all comparisons). Error bars represent standard deviations

Coronal plane alignment and joint space morphology were evaluated using two parameters: the femorotibial angle (FTA) and the medial joint space ratio (MJSR). FTA was measured as the angle between the anatomical axes of the femur and tibia on standing AP radiographs. Normal FTA in the general population ranges from 4° to 7° valgus; therefore, values below 5° were considered indicative of residual varus alignment. MJSR was calculated as the vertical height of the medial tibiofemoral compartment divided by the total tibial plateau width, providing a normalized index of medial joint space preservation (Fig. 4).

All radiographic assessments were performed by a blinded independent orthopedic researcher who was not involved in the surgical procedures.

### Statistical analysis

Although interim follow-ups at 2 and 6 weeks were conducted to monitor early recovery and complications, data from these timepoints were not included in the final analysis. Continuous variables were expressed as mean ± standard deviation, and categorical variables as percentages. Normality of continuous variables was assessed using the Shapiro–Wilk test. Parametric or non-parametric tests were selected accordingly. Within-group comparisons (pre- vs. postoperative) used paired *t* tests or Wilcoxon signed-rank tests. Between-group differences were analyzed using independent *t* tests for age and FTA, Mann–Whitney *U* tests for BMI, OKS, and MJSR, and chi-square tests for sex, side, and neurological events.

Two patients in each group (upper and lower PFO) underwent bilateral procedures. Each knee was treated as an independent unit in the analysis. Given the small and evenly distributed number of bilateral cases, and the absence of clustering in outcome patterns, no statistical adjustment for within-subject correlation was applied. This approach is consistent with prior orthopedic studies analyzing joint-level outcomes.

Cases with incomplete follow-up or missing outcome data were excluded using listwise deletion. Sample sizes for each analysis are reported in table footnotes where applicable. A *p* value < 0.05 was considered statistically significant. Analyses were conducted using SPSS Statistics v17.0 (IBM Corp., Armonk, NY, USA).

## Results

### Clinical outcomes

Of the 77 knees initially screened, 20 were excluded due to prior limb surgeries, neurological comorbidities, inadequate follow-up, or incomplete records, as illustrated in Fig. 1. The remaining 61 knees (57 patients) met the inclusion criteria and were included in the final analysis. There were no significant baseline differences between the upper and lower PFO groups regarding age, sex, BMI, surgical side, and OA grade (*p* > 0.05 for all; Table 1).

**Table 1:** Patient demographic characteristics

Variable	Upper PFO (n = 29)	Lower PFO (n = 32)	*p* value*
Age (years)	64.0 ± 9.5	62.6 ± 14.6	0.67
Gender (male:female)	10:19	10:22	‡0.94
Surgical side (left:right)	14:15	15:17	‡0.79
Body Mass Index (Kg/M^2^)	28.0 ± 3.23	28.9 ± 4.43	0.52
OA grading (II:III)^§^	16:13	17:15	‡0.83

*PFO* partial fibular osteotomy, *K–L* Kellgren–Lawrence

^‡^Chi-square test; §Radiographic grading of osteoarthritis; Grade IV excluded to focus on joints with structural preservation amenable to osteotomy

Both groups demonstrated statistically and clinically significant improvement in knee function and pain. In the upper PFO group, the Oxford Knee Score (OKS) improved from 23.17 ± 5.18 to 37.67 ± 3.26 (*p* < 0.001), and in the lower PFO group, from 27.00 ± 8.91 to 38.09 ± 6.04 (*p* < 0.001). A clinically meaningful OKS gain (≥ five-point improvement) was achieved in 93.1% and 87.5% of patients, respectively. This exceeds the MCID for OKS in knee OA (estimated at 4–5 points), supporting the clinical relevance of symptom improvement [22, 23].

Visual Analog Scale (VAS) pain scores improved from 5.14 ± 0.64 to 1.83 ± 0.84 in the upper PFO group, and from 5.07 ± 0.62 to 1.87 ± 0.76 in the lower PFO group (*p* < 0.001 for both). Postoperative scores did not differ significantly between groups (*p* = 0.83 for OKS, *p* = 0.75 for VAS).

At the 3-month follow-up, 92% of patients reported subjective improvement in knee function, consistent with the observed OKS gains. Perioperative metrics including operative time, time to ambulation, and hospital stay were comparable between groups, with the exception of slightly longer hospital stays in the upper PFO group (2.21 ± 0.6 vs. 1.98 ± 0.6 days, *p* = 0.04) (Table 2).

**Table 2:** Clinical and perioperative outcomes

Outcome	Upper PFO (n = 29)	Lower PFO (n = 32)	*p* value
Osteotomy level (cm) †	8.86 ± 1.64	14.8 ± 0.44	–
Segment length (cm) †	1.88 ± 0.26	1.68 ± 0.32	–
Operation time (min)	40.2 ± 6.3	39.1 ± 5.8	0.35
Time to ambulation (days)	0.61 ± 0.46	0.65 ± 0.39	0.75
Hospital Stay (days)	2.21 ± 0.6	1.98 ± 0.6	0.04
OKS—preoperative	23.17 ± 5.18	27 ± 8.91	0.01
OKS—3 months	37.67 ± 3.26	38.09 ± 6.04	0.83
VAS—preoperative	5.14 ± 0.64	5.07 ± 0.62	0.79
VAS—3 months	1.83 ± 0.84	1.87 ± 0.76	0.75
Drop Hallux (no.)	11	0	0.011 ‡
Numbness (no.)	9	0	0.031 ‡
Infection	Nil	Nil	1.00

*OKS* Oxford Knee Score (0–48), *VAS* Visual Analog Scale (0–10), *NS* not significant

^‡^Chi-square test; † Procedural parameters (osteotomy level, segment length) reflect protocol-defined differences and were not subjected to statistical comparison

Sample sizes reflect complete cases after listwise deletion

### Neurological and procedural safety

Neurological complications differed significantly between the two groups. A total of 11 cases (37.9%) of great toe weakness and 9 cases (31.0%) of dorsal foot numbness were observed exclusively in the upper PFO group, with no such deficits reported in the lower PFO group (*p* < 0.05). All deficits were mild to moderate in severity (manual muscle testing grades 3–4), with symptom onset occurring immediately postoperatively. Management included physical therapy, activity modification, and temporary ankle–foot orthosis (AFO) use in cases with gait instability. The majority of cases presented immediately postoperatively and demonstrated gradual improvement by the 3-month follow-up. Only a small number persisted beyond 8 months. All peroneal nerve palsies were transient and self-limiting, with complete recovery achieved at a mean of 8.25 ± 3.38 months (range 5–12 months). No surgical site infections or other major complications were recorded in either group (*p* = 1.00).

### Radiographic outcomes

Radiographic alignment, assessed via the femorotibial angle (FTA), showed small numerical improvements postoperatively in both groups; however, these changes were not statistically significant (*p* = 0.15; Table 3). Medial joint space ratio (MJSR) also remained stable with no significant postoperative variation (*p* > 0.05). Post hoc Pearson correlation analysis revealed no significant relationship between changes in FTA or MJSR and improvements in OKS or VAS (*r* = 0.12–0.18, *p* > 0.10 for all), suggesting that functional gains may be mediated by biomechanical or soft-tissue mechanisms beyond static alignment changes.

**Table 3:** Radiographic outcomes

Parameters	Upper PFO (n = 29)	Lower PFO (n = 32)	*p* value
FTA (°)—pre	2.89 ± 2.93	4.56 ± 3.28	0.02
FTA (°)—post	3.35 ± 1.99	4.80 ± 3.74	0.15
MJSR—preoperative	0.47 ± 0.32	0.40 ± 0.16	0.75
MJSR—postoperative	0.47 ± 0.34	0.41 ± 0.27	0.62

FTA was measured as the angle between the anatomical axes of the femur and tibia on standing AP radiographs; values < 5^0^ were considered indicative of residual varus alignment. MJSR was calculated as the vertical height of the medial tibiofemoral compartment divided by the total tibial plateau width

All values are expressed as mean ± standard deviation unless otherwise indicated

Sample sizes reflect complete cases after listwise deletion

*FTA* femorotibial angle, *MJSR* medial joint space ratio

**Fig. 5:**
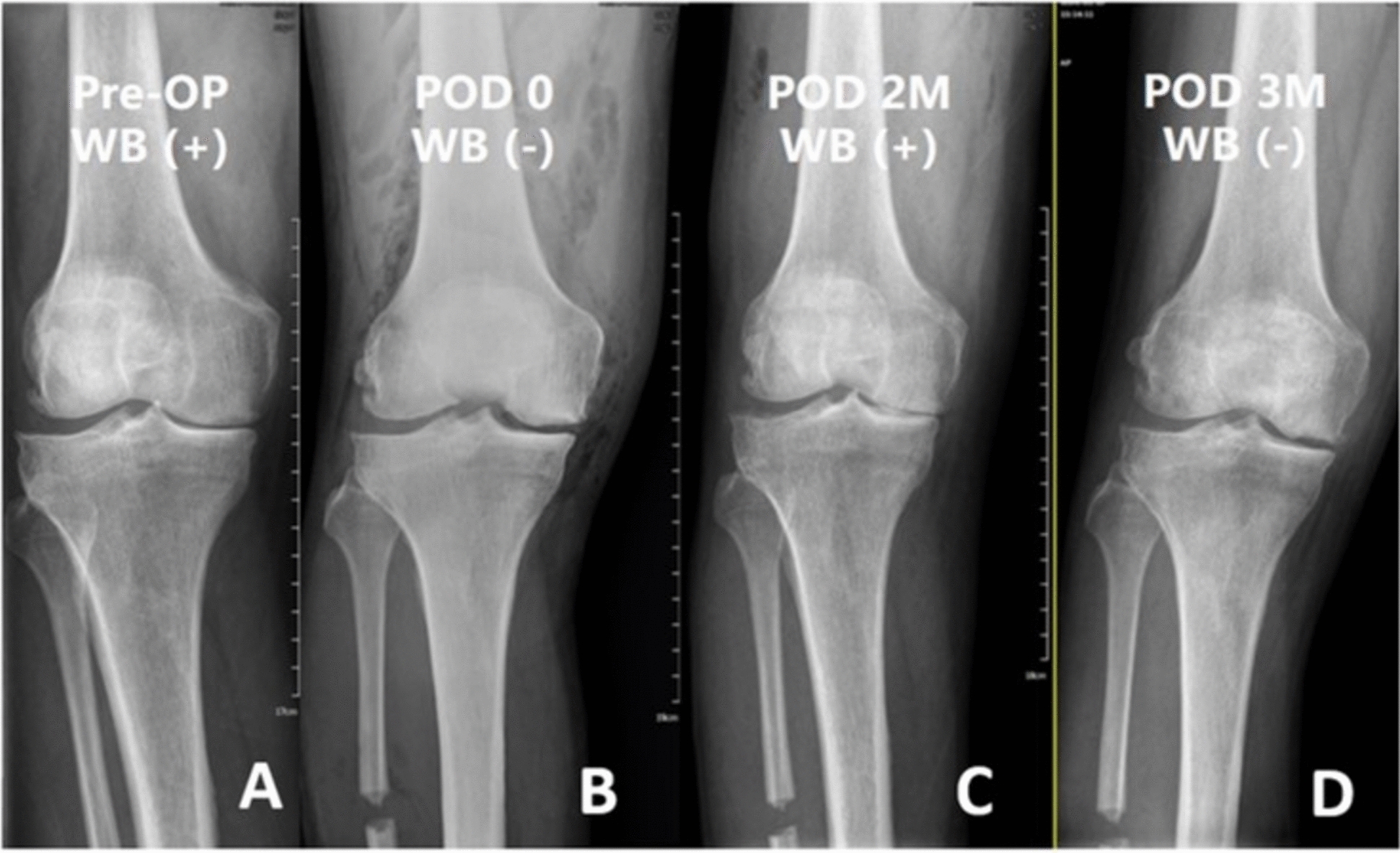
Serial radiographs illustrating the weight-bearing effect in medial joint space following PFO. **A** Preoperative standing radiograph [WB ( +)] shows marked medial joint space narrowing consistent with Kellgren–Lawrence Grade III osteoarthritis. **B** Immediate postoperative non-weight-bearing image [POD 0, WB (–)] reveals noticeable widening of the medial compartment. **C** Two-month postoperative standing radiograph [POD 2 M, WB ( +)] demonstrates persistent joint space narrowing under load, despite reported functional improvement. **D** Three-month postoperative non-weight-bearing image [POD 3 M, WB (–)] again shows medial joint space widening in the unloaded condition. These findings support the presence of dynamic medial compartment unloading that may not be fully appreciated on standard weight-bearing imaging. *WB* weight-bearing, *POD* postoperative day, *M* month

Although radiographic alignment changes were modest, functional gains were substantial, implying that symptomatic improvement may be mediated through mechanisms other than coronal correction alone. Notably, medial joint space widening was more apparent in postoperative non-weight-bearing radiographs compared to weight-bearing images (Fig. 5), suggesting that unloading of the medial compartment may not be fully reflected in standard standing films.

## Discussion

This study demonstrates that lowering the fibular osteotomy level to the mid-diaphysis (12–15 cm below the fibular head) significantly reduces the incidence of peroneal neuropathy—from 30 to 0%—while preserving clinically meaningful improvements in pain and function. These findings directly confirm our hypothesis and suggest that modifying the PFO technique enhances both neurological safety and therapeutic consistency in treating medial compartment knee osteoarthritis (MKOA).

From a cost-effectiveness perspective, PFO offers compelling advantages over traditional alternatives. In our cohort, operative time averaged 40 min, patients ambulated within 24 h, and hospital stays lasted approximately 2 days, compared to reported averages of 90–120-min operative time, 5–7-day hospitalization, and extended rehabilitation timelines for high tibial osteotomy (HTO) or total knee arthroplasty (TKA) [1, 24]. In addition to early ambulation, most patients resumed unassisted walking within 3–5 days and returned to routine daily activities within 2–3 weeks. These recovery metrics compare favorably to HTO, which typically requires 4–6 weeks of protected weight-bearing, and TKA, which often involves 6–8 weeks of structured rehabilitation [24, 25]. Compared to unicompartmental knee arthroplasty (UKA), which requires implant-specific instrumentation and longer rehabilitation, PFO offers a simpler, implant-free alternative. Arthroscopic debridement, while minimally invasive, has shown limited long-term benefit in MKOA and lacks the unloading effect achieved by PFO.

Importantly, 93% of upper-level and 87.5% of lower-level PFO patients achieved a ≥ five-point improvement in Oxford Knee Score (OKS), surpassing the established minimal clinically important difference (MCID) of 4–5 points [22, 23]. This supports the clinical relevance of the functional outcomes reported. In addition, Visual Analog Scale (VAS) scores declined by over 60% postoperatively in both groups, reinforcing consistent symptom relief despite different osteotomy levels. Patient-reported satisfaction was also high: 92% of patients reported improved function at 6 months, and informal feedback indicated strong acceptance of the procedure, particularly in the lower PFO group, where no neurological complications were observed.

The absence of complications in the lower PFO group is striking. In contrast, 37.9% of upper-level knees experienced great toe weakness, and 31% developed dorsal foot numbness, likely reflecting the proximity of the osteotomy site to the superficially coursing common peroneal nerve and its branching points. Anatomical studies have identified the 6–10 cm region below the fibular head as a neurologically hazardous zone [19, 20], consistent with our complication profile. All nerve symptoms were transient and resolved within 12 months, but their incidence justifies considering surgical modification as a patient safety imperative. This decision was ethically grounded: after observing a high rate of early complications, we revised the technique to prioritize patient safety without compromising efficacy. This reflects a core principle of surgical ethics—continuous refinement in response to observed harm.

The clinical benefits of PFO, particularly at mid-fibular levels, appear to occur without substantial changes in radiographic alignment or joint space measurements. While femorotibial angle (FTA) and medial joint space ratio (MJSR) showed non-significant postoperative shifts (Table 3), symptomatic relief was robust. Post hoc correlation analysis further confirmed that changes in FTA and MJSR were not significantly associated with improvements in OKS or VAS, suggesting that functional gains may be mediated by biomechanical or soft-tissue mechanisms beyond static alignment. This supports the “arch-string theory,” which posits that fibular resection decreases lateral column tension—altering soft tissue biomechanics and redistributing load across the tibial plateau [9, 10]. Finite element analyses by Kang et al. further demonstrated reduced medial compartment stress with fibular resection at varying levels, without major changes in frontal plane alignment [8].

An intriguing observation was the widening of medial joint space on non-weight-bearing radiographs postoperatively (Fig. 5), contrasting with persistent narrowing under load. This may reflect dynamic decompression not captured by static standing films, suggesting a novel mechanism of action. Future research incorporating gait analysis or dynamic stress imaging could clarify how fibular osteotomy influences joint loading patterns throughout the gait cycle.

While literature has inconsistently reported radiographic changes following PFO—Huang et al. observed alignment shifts, whereas Huda and Sabir found none [14, 26, 27]—our data align with the latter group, possibly due to our use of early weight-bearing protocols and mid-shaft osteotomy levels. This highlights the need for standardized techniques and comparative biomechanical validation.

It should be noted that our study population included a relatively broad demographic range, with patients aged 45–78 years, BMI between 22 and 32, and radiographic severity limited to K–L grade II or III. While this introduces some heterogeneity, it may enhance the generalizability of our findings to real-world clinical settings.

This study does have limitations. Patients were allocated chronologically, not randomly, to upper or lower PFO, introducing potential selection bias. However, baseline demographics were statistically equivalent, and all procedures were performed by the same senior surgeon with > 20-year experience, minimizing variation. Nonetheless, chronological allocation may have introduced unmeasured confounders such as seasonal variation in rehabilitation access, evolving institutional protocols, or subtle shifts in patient selection criteria over time. While these factors were not quantifiable in our data set, they represent potential sources of bias that should be addressed in future randomized studies. In addition, the transition to a lower osteotomy level introduced a procedural change that may have been subject to a learning curve. Although the surgeon was highly experienced, early cases in the lower PFO group may have benefited from increased familiarity with the modified technique, potentially influencing complication rates or operative efficiency. In addition, technical nuances such as fibular segment length (typically 1.5–2 cm in our series), surgical approach (posterolateral), and soft tissue handling may influence both efficacy and complication rates. Future studies should explore whether these variables affect outcomes independently of osteotomy level.

In the absence of significant coronal realignment, the long-term durability of symptom relief remains uncertain. Without mechanical axis correction, some patients may experience recurrence of symptoms due to progressive joint degeneration. While the preservation of native joint structures and avoidance of implants are theoretically advantageous, these potential benefits remain speculative. To date, no mid-to-long-term survival data have been reported for conventional or modified PFO techniques. These uncertainties underscore the need for prospective longitudinal studies to evaluate durability, structural progression, and the role of PFO in long-term treatment algorithms.

## Clinical implications and future directions

This study supports mid-diaphyseal PFO as a technically accessible, neurologically safer, and economically viable option for patients with medial compartment knee osteoarthritis—particularly those who are not candidates for implant-based procedures. Its minimal invasiveness, short recovery timeline, and low complication rate make it well-suited for broader adoption in both high-volume and resource-limited settings.

Looking ahead, several priorities emerge:

- **Prospective validation**: Randomized trials are needed to confirm the safety and efficacy of mid-level PFO and to control for potential selection bias and learning curve effects.
- **Biomechanical modeling**: Musculoskeletal simulations and gait analysis could clarify how fibular osteotomy alters load distribution and joint mechanics in real time.
- **Long-term surveillance**: Extended follow-up is essential to determine whether symptom relief is durable in the absence of mechanical axis correction.
- **Patient-centered outcomes**: Incorporating validated PROMs and satisfaction metrics will help define which subgroups derive the greatest benefit.
- **Technique refinement**: Future studies should explore how variables, such as segment length, surgical approach, and soft tissue handling influence outcomes.

Ultimately, mid-level PFO may represent a paradigm shift in the treatment of MKOA—offering a low-risk, high-value alternative that bridges the gap between conservative care and joint replacement.

## Conclusion

Lowering the fibular osteotomy level to the mid-diaphysis effectively eliminated peroneal neuropathies, reducing the complication rate from approximately 30% to 0% and significantly enhancing the neurological safety of the procedure. This technical modification preserved the clinical benefits of pain relief and functional improvement while avoiding major nerve-related complications, thereby improving the overall risk–benefit profile of PFO. As a proposed alternative to high tibial osteotomy (HTO) and total knee arthroplasty (TKA), PFO offers simplicity, rapid recovery, and lower cost—estimated at 30–40% of traditional procedures—making it particularly valuable in resource-limited settings or for patients unsuitable for implant-based interventions. The mid-diaphyseal approach introduced in this study further strengthens the safety and consistency of PFO, reinforcing its feasibility and clinical utility.

These findings support the adoption of mid-diaphyseal fibular osteotomy as the preferred technique when performing PFO for patients with MKOA. Given the retrospective design and limited follow-up, further prospective studies are needed to assess long-term durability, define optimal patient selection, and clarify the role of PFO within the broader treatment landscape for medial knee osteoarthritis.

## Data Availability

All data can be accessed by contacting the corresponding author with a justified request.

## Abbreviations

MKOA: Medial knee osteoarthritis
PFO: Partial fibular osteotomy
VAS: Visual Analog Scale
OKS: Oxford Knee Score
FTA: Femorotibial angle
MJSR: Medial joint space ratio
HTO: High tibial osteotomy
TKA: Total knee arthroplasty
